# Scaling and Structural Properties of Juvenile Bull Kelp (*Nereocystis luetkeana*)

**DOI:** 10.1093/iob/obab022

**Published:** 2021-08-14

**Authors:** Katie A Dobkowski, Stephanie B Crofts

**Affiliations:** Department of Biology, Bates College, 44 Campus Ave, Lewiston, ME 04240, USA; Friday Harbor Laboratories, University of Washington, 620 University Road, Friday Harbor, WA 98250, USA; Department of Biology, University of Washington, 24 Kincaid Hall, Box 351800, Seattle, WA 98195, USA; Friday Harbor Laboratories, University of Washington, 620 University Road, Friday Harbor, WA 98250, USA; Department of Biology, College of the Holy Cross, 1 College Street, Worcester, MA 01610, USA

## Abstract

Bull kelp (*Nereocystis luetkeana*), the only canopy-forming kelp in the Salish Sea, provides primary production in the nearshore subtidal environment and serves as an important habitat for economically and ecologically important species. An annual species, each year juvenile bull kelp sporophytes must grow from the hydrodynamically more benign benthos to the water column, where they experience substantial drag at the surface. Because of the differences in morphology and ecology across life stages, and the fact that previous work has focused mainly on adult bull kelp, we tested whether morphology and structural properties change with stipe length, investigating scaling of both juvenile (stipe length < 40 cm) and mature (stipe length > 40 cm) kelp, and testing how juvenile stipes fail. Juvenile bull kelp grow proportionally (isometric growth) when young, but lengthen more quickly than would be predicted by bulb size (negative allometry) at maturity. Based on our data, the predicted breakpoint between isometric and allometric growth occurred at about 33 cm, likely approximately one to two weeks of growth. Cross-sectional area of the stipe, force to failure, work to failure, and stiffness (Young's modulus) all grow more slowly than would be predicted based on length, while maximum stress and toughness increase more quickly than predicted. There is no change in extensibility over the size range we tested, suggesting that this material property does not change with stipe length. The differences in biomechanics between juvenile and adult kelp are likely a response to the varied hydrodynamic environments experienced during the annual life cycle, which highlights the importance of studying organisms across life stages.

## Resumen

*Nereocystis luetkeana* este singura formă de varec în Marea Salish care face baldachin și care furnizează producția primară în mediul submareal din apropiere și creează habitat important pentru specii importante din punct de vedere economic și ecologic. În fiecare an sporofiții juvenili *N. luetkeana* trebuie să crească din bentosul hidrodinamic mai benign până la coloana de apă, unde experimentează o rezistență substanțială la suprafață. Datorită diferențelor dintre morfologie și ecologie de-a lungul etapelor vieții, și faptului că lucrările anterioare s-au concentrat în principal pe *N. luetkeana* adulte, am testat dacă morfologia și proprietățile structurale se schimbă odată cu lungimea tulpinei investigând scalarea atât a puietului juvenil (lungimea stipei < 40 cm), cât și a celui matur (lungimea tulpinei > 40cm), și testând modul în care tulpinile juvenile eșuează. *Nereocystis luetkeana* juvenilă cresc proporțional (creștere izometrică) când sunt tineri, dar se prelungesc mai repede decât s-a prevăzut de mărimea bulbilor lor (alometrie negativă) când sunt maturi. Pe baza datelor noastre, punctul de rupere prevăzut între creșterea izometrică și alometrică s-a produs la aproximativ 33 cm, care a fost probabil de aproximativ 1–2 săptămâni de creștere. Secțiunea transversală a tulpinii, Forțarea de a eșua, munca până la eșec, rigiditate (modulul lui Young) toate se dezvoltă mai încet decât ar fi de așteptat pe baza lungimii, în timp ce stresul și rezistența maximă cresc mai repede decât este prevăzut. Nu există nicio modificare a extensibilității în intervalul de mărimi testat, ceea ce sugerează că această proprietate materială nu se modifică odată cu lungimea tulpinei. Diferențele în biomecanică între *N. luetkeana* juvenilă și adultă sunt probabil un răspuns la mediile variate hidrodinamice pe care acestea le experimentează în timpul ciclului de viață anual, ceea ce evidențiază importanța studierii organismelor de-a lungul etapelor vieții.

## Introduction

**Fig. 1:**
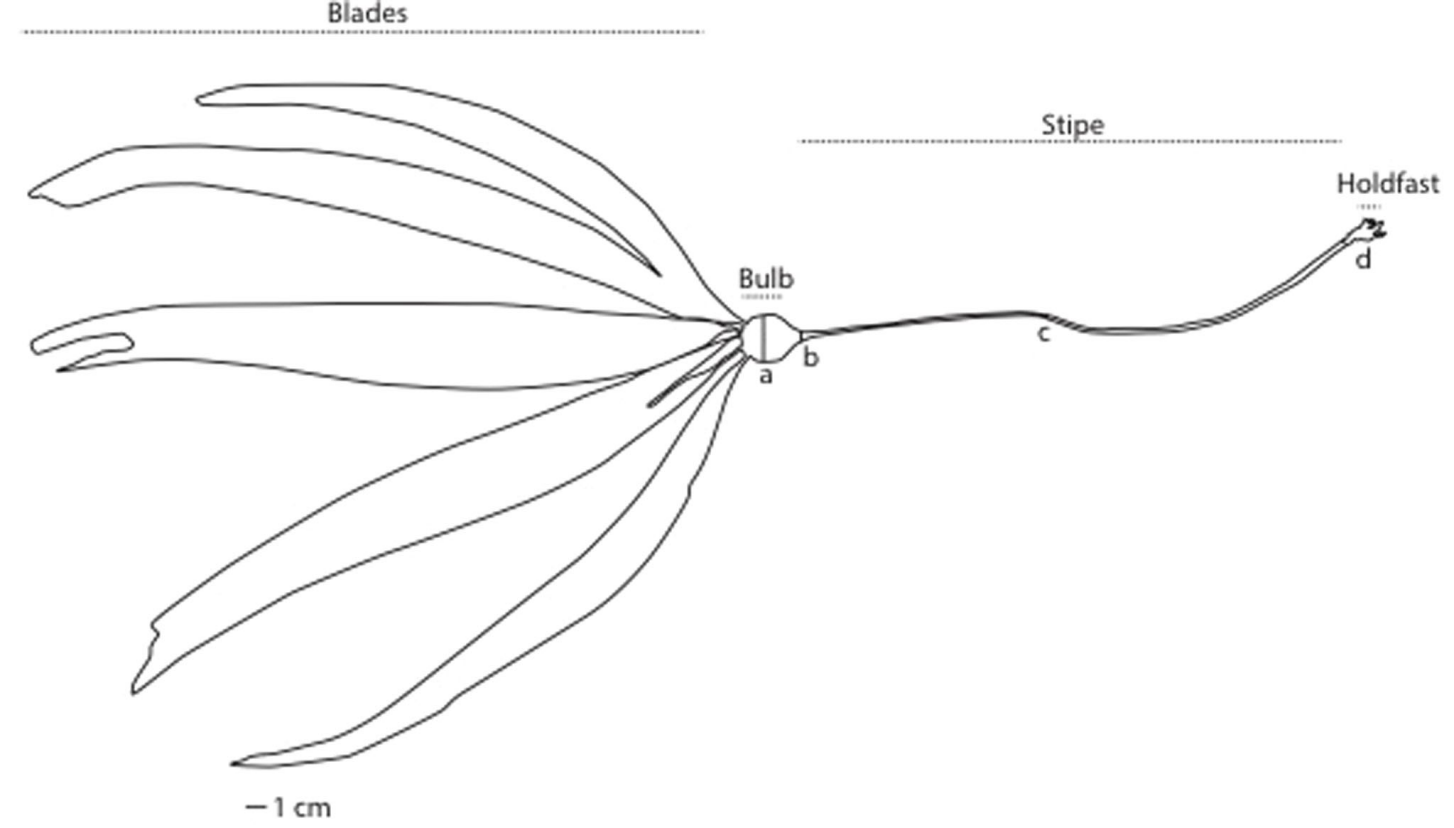
Bull kelp morphology (dotted lines) and morphological measurements (solid lines; a = bulb width, b = stipe width underneath the bulb, c = narrowest stipe width, d = stipe width above holdfast).

Bull kelp, *Nereocystis luetkeana* (K.Mertens) Postels and Ruprecht 1840, is an important foundation species in the nearshore subtidal zone of the Salish Sea (Dayton 1985). Kelp beds are formed by multiple individual sporophytes, each consisting of a holdfast attached to the benthos, a stipe that can reach up to 30 m in length, and a floating bulb supporting up to 100 blades (Fig. 1; Foreman 1976). It is a primary producer in shallow water, and subsidizes food webs below the photic zone, as detached stipes and blades are eventually transported to the benthos (Duggins et al. 1989; Britton-Simmons et al. 2009). Like all kelp (Laminariales), bull kelp exhibits a heteromorphic life history with alternating macroscopic sporophyte and microscopic gametophyte stages (John 1994). Unlike many other species, bull kelp is an annual species, and individuals must successfully complete all life stages within a single growing season for bull kelp beds to persist from year to year (Scagel 1947).

Hydrodynamic forces that the growing kelp sporophyte encounters will differ near the substrate and at the surface. The presence of a boundary layer very close to the substrate where flow is nonexistent is the most dramatic example, but more of a concern for the microscopic gametophyte life stage. For the growing sporophyte, the conditions above the boundary layer (still far from the surface) compared with at/near the water surface are also very different and may display local variability based on unique topography (Eckman et al. 2003). At depth, the main hydrodynamic forces would be current-driven flow, while at the surface, tidal currents and wind-driven currents may act in combination or in opposition depending on local conditions. For example, even in the case of a hypothetical storm-generated wave, the maximum horizontal current speed at depths between 10 and 20 m would not exceed 100 cm s^−^^1^, but would increase as high as 700 cm s^−1^ by 2 m depth (Eckman et al. 2003).

Bull kelp also exhibit high levels of morphological plasticity that can influence the shape of different structures (like the blade) based on local hydrodynamic forces, which can, as a result, affect the forces applied to the tissues. For example, adult bull kelp growing in slower flow environments frequently exhibit a “ruffled” blade morphology, caused by differences in growth rates, that would create drag on the blades and increase the likelihood of damage in faster flow environments; blades experiencing unidirectional flow can still grow large by reconfiguring (for example, by clumping together to increase the ability to streamline) (Koehl and Alberte 1988; Koehl and Silk 2021). When growing tissue is subjected to tensile force, bull kelp blades grow longer, narrower, less ruffled, and heavier (Coleman and Martone 2020). Growing in a dense group (“bed”) with conspecifics helps moderate the water flow within the interior of the bed, enabling individual bull kelp growing in that specific area to maintain larger blade sizes (Gaylord et al. 2007). Hydrodynamic forces, while most obviously affecting blade morphology, can also affect facets of bull kelp stipe morphology, such as diameter (Johnson and Koehl 1994).

In addition to more variable current conditions and increasing flow velocity and drag in the water column, mature canopy-forming kelp must cope with tangling with conspecifics, holdfast detachment, as well as damage from consumers. Environments with variable current flow especially increase the risk of adult bull kelp mortality and even a small amount of damage can greatly reduce the force required to break the stipe (Duggins et al. 2001). Consumers such as isopods (genus *Idotea*), red sea urchins (*Mesocentrotus franciscanus*), and kelp crabs (genus *Pugettia*) readily consume and damage fresh and/or detrital bull kelp (Dethier et al. 2014; Dobkowski 2017). Tangling can also contribute to bull kelp failure, as it does in giant kelp, *Macrocystis pyrifera* (Seymour et al. 1989; Burnett and Koehl 2018). Undamaged bull kelp stipes may fail if detached individuals become tangled with them, or when neighboring individuals become entangled, both of which will increase drag and exert greater/unequal force on individual holdfasts (Koehl and Wainwright 1977).

The young, fast-growing bull kelp sporophytes likely face a different set of challenges than adults. One of those challenges is light availability; other canopy-forming seaweeds, such as the giant kelp (*M. pyrifera)*, have been shown to reduce available bottom light by 60% as compared with levels at the surface (Clark et al. 2004). The young bull kelp sporophyte may face fewer hydrodynamic challenges than adults, as there is slower, more unidirectional water flow near the benthos than higher up in the water column, but they are certainly subject to attack by diverse and/or abundant herbivores. Juvenile bull kelp grow more when they are caged to exclude large consumers such as the Northern kelp crab (*Pugettia producta*) or sea urchins (*Strongylocentrotus, Mesocentrotus*) indicating some level of top-down control of this life stage by herbivores (Dobkowski 2017). Even small snails (*Lacuna vincta*) can be a threat—field observations show greater snail density and stipe damage on juvenile bull kelp than adult conspecifics (Chenelot and Konar 2007). Similarly, damage by small, herbivorous amphipods contributed to *Lessonia berteroana* stipe failure, leading to significant breakage and tissue loss (Gutow et al. 2020). As with adult kelp, damage will change the cross-sectional area of the stipe, concentrate stress on that point, and make failure there more likely (Koehl and Wainwright 1977). Differences in tidal currents may contribute to differences in herbivore abundance, with strong tidal currents leading to lower prevalence of grazers like *L. vincta* (Eckman et al. 2003).

These various biotic and abiotic threats to bull kelp vary with size, and may affect growth. Modeling experiments suggest that smaller (<1 m) kelp may grow quickly in stipe length and proportionally to bulb width (isometrically) to contend with large stresses generated by drag on the blades as they enter the water column, but grow allometrically (stipes lengthen at a faster rate than the rate at which bulbs increase in circumference) at larger sizes because their size allows them to mediate the effects of wave-driven currents by “going with the flow” (Denny et al. 1997). Blade mass or frond area in mature subtidal and intertidal kelp scales with negative allometry when compared with stipe mass, which will variably affect the amount of drag on the kelp and change the way individuals interact with the environment (Friedland and Denny 1995; Starko and Martone 2016).

Growth itself also affects material properties of macroalgae and will impact stipe failure, and different regions of kelp may grow at different rates. For non-canopy-forming kelp, material properties change relative to distance from the meristem: newer, younger blades tend to be less stiff and more extensible than older tissues (Krumhansl et al. 2015). *Neoagarum* and *Saccharina*, both of which remain closer to the less turbulent benthos, exhibit unidirectional growth, where the youngest, most extensible tissue is near the holdfast, and the older, stiffer tissue sloughs off at the distal end of the blade (Mann 1973; Krumhansl et al. 2015). *Egregia menziesii* rachis tissue stiffness, extensibility, and strength also vary with distance from the meristem, and this difference is compounded by variable growth rates, such that slower growing tissue will be overall older, stiffer, less extensible, and stronger than more quickly growing tissues (Burnett and Koehl 2019). Mature bull kelp follow a similar pattern; the narrow region just above the holdfast (Fig. 1D), where rapid growth takes place in young individuals, is highly extensible and tough compared with the oldest hollow region of the stipe, closest to the surface (Fig. 1B; Koehl and Wainwright 1977). When juvenile bull kelp are far from the surface, they grow primarily in stipe length; once they are near the surface, the intercalary meristem between the bulb and blades becomes most active, enabling bifurcation and proliferation of blades to capture light for photosynthesis.

Because both material properties and structure contribute to stipe integrity, it is important to understand how these vary with life stage. Bull kelp serves as a tractable system in which to study this variation of material and structural properties with life history, because it is an annual species. In order for the adult sporophyte to reach the surface and achieve reproductive maturity, it must successfully grow past the juvenile stage, where individuals extend less than a half a meter from the substrate. The goals of this study are to (1) determine how juvenile bull kelp scale, particularly the relationship between bulb size and stipe length; (2) identify differences in stipe failure over a range of sizes, again using stipe length as a proxy for position in the water column; and (3) compare the morphology and material properties of juvenile and adult bull kelp, with focus on very young sporophytes from a week (2 cm) to several weeks old (36 cm).

The growth rates of small bull kelp may vary widely due to environmental factors like availability of light, making it hard to determine an “age”. We are particularly interested in how these various material and structural properties change as stipes grow up through the different flow environments of the water column, and would expect to see some combination of morphological and material properties modification with increased stipe length. Blade area is important for understanding hydrodynamic forces, but small bull kelp blades reliably show evidence of extensive and variable damage (both mechanical and by consumers), making it hard to use this as a reliable measure (Dobkowski 2017). Because of this, we focus our measurements on stipe morphology (compared with bulb width) and material properties. Kelp may respond to increased flow by increasing their extensibility (making it easier to reconfigure), by making an overall wider stipe, or by increasing the overall toughness of their stipe material (requiring more energy to fail). To see whether these changes occur, we measured strain at failure (ε_fail_), maximum stress (σ_max_), and Young's modulus, as well as the overall force (F) and work (W) required to induce failure in the stipes.

## Methods

### Field collection

**Fig. 2:**
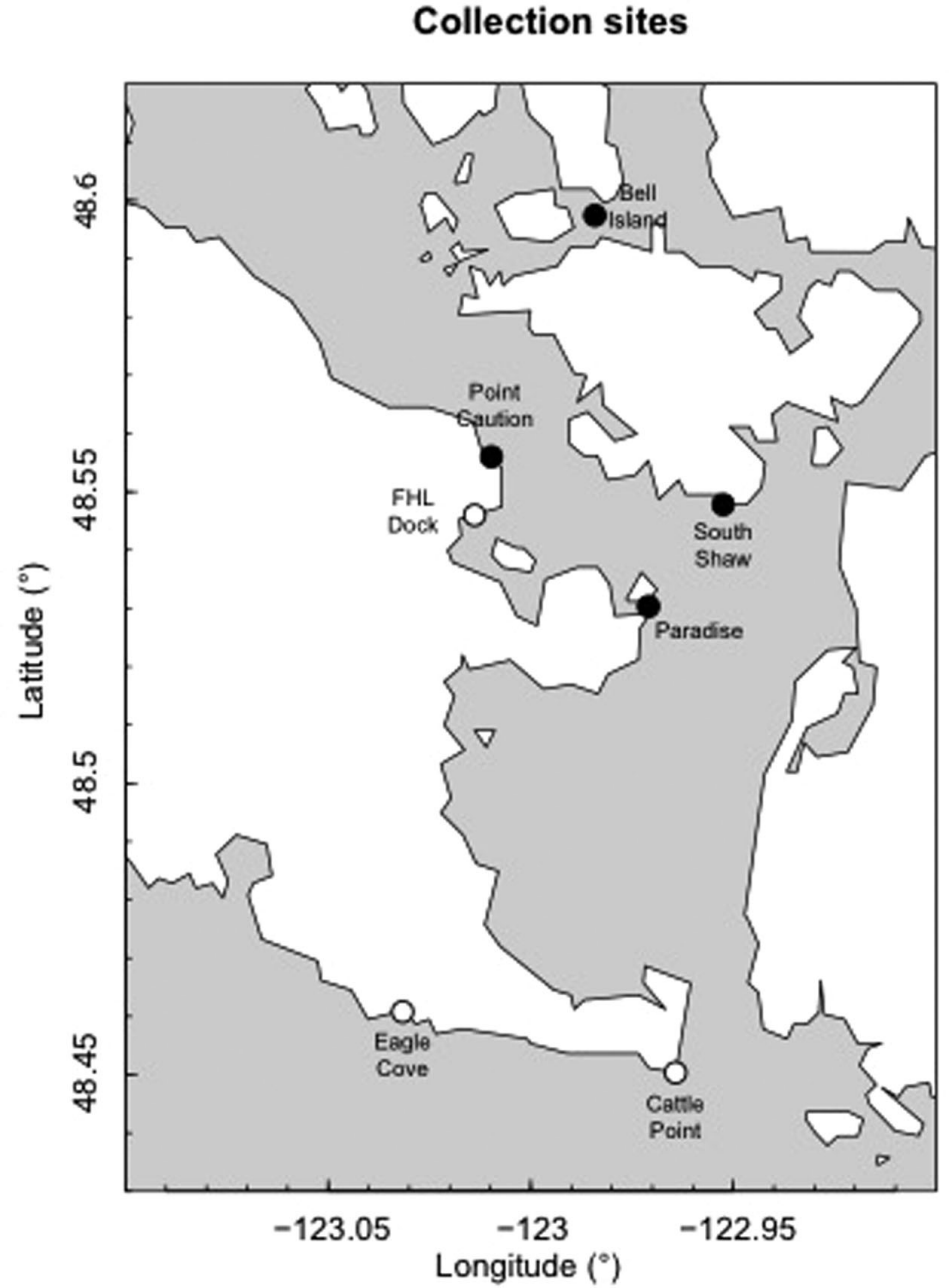
Map of kelp collection sites along the San Juan Channel near Friday Harbor, WA. Empty dots represent sites where adult drift kelp were collected. Solid dots indicate sites where subtidal juvenile kelp were collected.

We collected 28 juvenile bull kelp for materials testing (stipe lengths 2.1–36.1 cm) at three subtidal sites in the San Juan Channel near Friday Harbor Laboratories (Fig. 2): South Shaw (48**°**33′12.33″N; 123**°**0′26.00″W), Paradise (48**°**31′42.47″N; 122**°**58′15.71″W), and Bell Island (48**°**35′42.38″N; 122**°**58′45.75″W). All of these sites are moderately protected from direct wave activity; because of their relative locations in or adjacent to the San Juan Channel, these sites also experience similarly strong tidal currents (Duggins et al. 2001).

Using dive knives, scuba divers removed intact holdfasts from the substrate without applying force to the stipe or the junction between the stipe and the holdfast, and we maintained the juvenile kelp individuals in flow-through seawater tables until materials testing took place. To minimize specimen degradation, we tested individuals within 48 h of collection and measured and photographed each kelp prior to testing. We recorded stipe length (mm), bulb width (mm), stipe width below the bulb and above the holdfast (mm), as well as the region of the stipe and stipe width where the stipe was visually estimated to be narrowest (mm; Fig. 1).

Additionally, we collected 27 intact, mature drift bull kelp (i.e., detached individuals floating at the surface) at three sites on San Juan Island: the FHL dock (48**°**43′42.14″N; 123**°**00′44.02″W), Eagle Cove (48**°**27′41.45″N; 123**°**01′55.18″W), and Cattle Point (48**°**27′00.06″N; 122**°**57′45.94″W). For these larger, mature individuals, we measured the stipe length and bulb diameter to allow for comparison of morphological scaling relationships to the juvenile kelp. Because we had no control over the state of degradation in these individuals and did not know from which specific kelp bed locations they originated, their material properties were not tested but this haphazard sampling decision did provide a broad range of varied adult bull kelp morphology.

### Lab testing

**Fig. 3:**
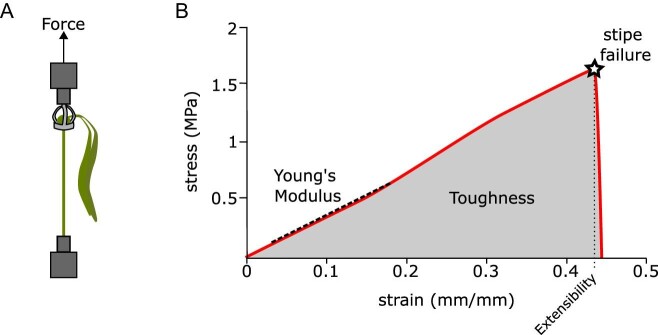
Experimental setup and sample results. **(A)** Diagram showing kelp holdfast freeze-clamped to metal clamp, bulb held in 3D printed kelp cradle, and cradle held by clamp attached to moving crosshead and force transducer. **(B)** Sample stress–strain curve showing Young's modulus (tangent slope), extensibility (ε_fail_), and toughness (area under the curve).

To measure the material properties of juvenile kelp, we loaded each individual in tension using a materials testing system (MTS; Synergie 100, MTS Systems Corp., Eden Prarie, MN) fitted with a 500 N load cell recording at 100 Hz. We used four custom 3D printed cradles to pull kelp stipes while minimizing stress concentrations on the bulbs (Fig. 3A). If a kelp bulb was too small to fit in a cradle, we freeze-clamped the bulb in place by loosely clamping the tissue with metal clamps and applying dry ice to the clamps, effectively freezing the tissue in place. Depending on the size of the individual kelp, we used a similar 3D printed platform or freeze-clamped the holdfast to a metal clamp to keep them stationary (Fig. 3A). For each test, we loaded the stipes by raising the bulb at a rate of 50.8 mm min^−1^ until the stipe failed. Prior to data collection, we ran pilot tests to determine the strain rate that would be most effective for the experiment, allowing for accurate and repeatable data collection. If failure occurred at the clamp or within the cradle, we assumed this was due to artificially high stress concentrations and excluded the specimen from analysis. In some cases, stipe length exceeded the working height of the MTS (stipe lengths exceeding ∼22 cm; *n* = 3 individuals), so to ensure kelp were subjected to a purely vertical tensile load, we used a pulley system in conjunction with the MTS to test longer specimens. For each specimen tested, we took photographs of the fractured stipe surface, which were analyzed in ImageJ (NIH, Version 1.51f) to determine the cross-sectional area of the break.

We collected force-extension data for each specimen (to the nearest 0.01 N and 0.00001 mm, respectively), and recorded the maximum force (F_Max_; N), the force at failure (F_fail_; N) defined as a 50% drop in force, and work to failure (W_fail_, J). All three of these measures will be influenced by both the material properties of the stipe, and stipe size and shape. To isolate the material properties from the morphology, we used fracture surface area to generate stress–strain curves (Fig. 3B) for each test. In our experiments, stipe breaks were smooth and perpendicular to the long axis of the stipe, so the cross-sectional area of the stipe at the point of fracture was functionally equivalent to the fracture surface area and specific to each kelp stipe tested. We calculated stress by normalizing the applied force by the fracture surface area (σ = F/SA), and calculated conventional strain as the ratio of the change in stipe length to the initial length. For each experimental kelp pull, we used these calculations to determine conventional strain at failure (ε_fail_), maximum stress (σ_max_, Pa), Young's modulus (Pa), and toughness (work of extension to failure, MJ m^−^^3^). Conventional strain at failure (ε_fail_) is a measure of how extensible the stipe material is, Young's modulus is a measure of material stiffness, and toughness is a measure of the energy absorbed by the stipe material (Fig. 3B). These values describe the material properties of the stipe, independent of the morphology.

### Data analysis

We analyzed our data with R (R Core Team 2016; R version 3.3.2 [2016-10-31]—“Sincere Pumpkin Patch”) using the “lmodel2” package to perform Model II regressions (on log-transformed values) using the standard major axis method when both variables were random and subject to measurement error that needed to be minimized (Legendre 2014). For ε_fail_, we used the ordinary least squares method for our regression, because this represents a unit-less calculated value. We used the R package “segmented” to predict the “breakpoint” between juvenile and adult modes of scaling (Muggeo 2008).

Our null hypothesis (*H*_0_) assumes no difference in material properties as kelp grow (i.e., between small bull kelp closer to the substrate and mature kelp that have reached the surface), and isometric scaling. Therefore, our predicted slopes describing the (log-transformed) relationships between stipe length and Young's modulus, σ_max_, ε_fail_, and toughness will all be zero, as these material property measures should be independent of morphology. We predict that our comparison of the log-transformed adult and juvenile stipe lengths versus bulb widths will have a slope of 1, meaning under our null hypothesis the stipes and bulbs will grow at the same rate. We predict that under our null hypothesis, the comparison of log-transformed force versus stipe length will have a slope of 2, as the force required to induce failure should scale with the cross-sectional area over which it is applied. Finally, for our comparison of W_fail_ versus stipe length, we predict that under the null hypothesis our log-transformed data will show a slope of 3, as W_fail_ will scale with volume (assuming the shape of the stipe is an approximate cylinder) (Table 1). We inferred allometric scaling (positive or negative) if the predicted isometric slope fell significantly outside the calculated 95% confidence intervals of the experimental slope.

**Table 1:** Scaling analyses of morphological (adult, juvenile) and biomechanical properties (juvenile) of bull kelp

Regression	*H*_0_	Slope	±95% CI	Type of scaling*	*y*-intercept	*R* ^2^
*Adult Kelp*						
Bulb∼Stipe	*b* = 1	0.70	0.54–0.91	Negative allometry	−2.44	0.59
*Juvenile Kelp*						
Bulb∼Stipe	*b* = 1	1.19	0.96–1.49	Isometric	−3.49	0.78
BreakSurfaceArea∼Stipe	*b* = 2	0.66	0.49–0.94	Negative allometry	−1.98	0.41
Young's modulus∼Stipe	*b* = 0	−1.23	−(1.44)–(−1.05)	Negative allometry	7.61	0.88
MaxStress∼Stipe	*b* = 0	0.55	0.36–0.87	Positive allometry	−2.23	0.03
ConventionalStrainFail∼Stipe	*b* = 0	0.02	−0.17–0.21	Independent	0.67	0.003
Toughness∼Stipe	*b* = 0	1.31	0.98–1.75	Positive allometry	−7.03	0.61
F_fail_∼Stipe	*b* = 2	0.69	0.51–0.95	Negative allometry	−1.71	0.55
Work∼Stipe	*b* = 3	1.80	1.44–2.26	Negative allometry	−4.25	0.76

“Stipe” refers to stipe length; *H*_0_ = null hypothesis; CI = confidence interval, used to determine whether slope varies from isometric.

*Scaling relative to the null hypothesis.

## Results

Slope, intercept, *R*^2^, and confidence interval values for all comparisons are summarized in Table 1.

### Morphology

**Fig. 4 (A):**
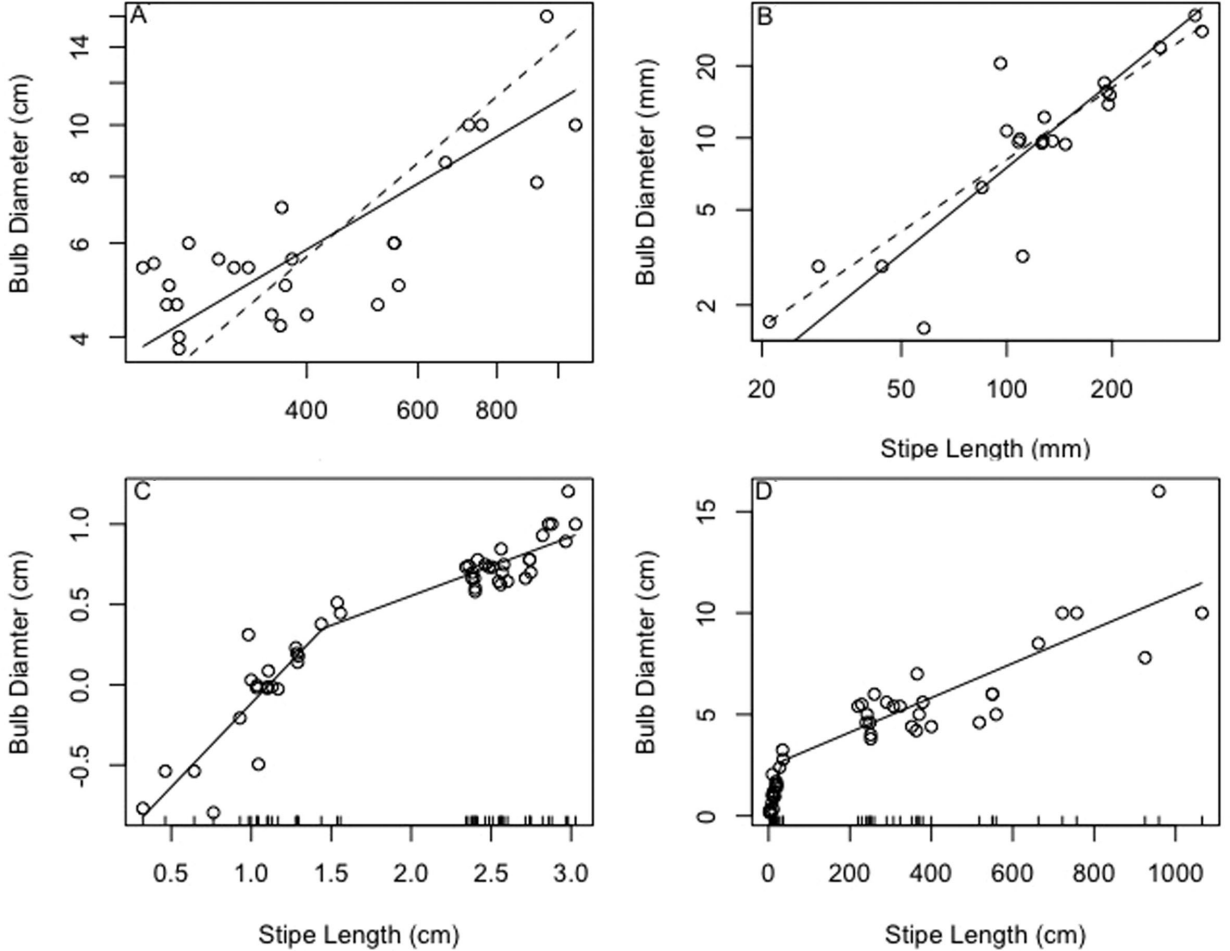
Mature bull kelp (*n *= 27) bulbs show negative allometry with stipe length as they grow; **(B)** juvenile bull kelp (*n *= 22) bulbs scale isometrically with stipe length. Solid lines show standard major axis regression lines; dotted lines show predicted isometry (predicted slope = *H*_0_ = 1). **(C)** Log-transformed data showing transition (breakpoint) in scaling between small juvenile (isometric) and large adult (allometric) kelp. **(D)** Growth switches from isometry to allometry at a predicted stipe length of 33.2 cm (±14.8 SE).

For mature bull kelp (stipe length > 200 cm), our data show negative allometry, meaning that stipe length increases more rapidly than the bulb width (slope = 0.71; Fig. 4A). In contrast, juvenile bull kelp (length < 40 cm) scale isometrically (slope = 1.19; Fig. 4B). There is a breakpoint where this relationship changes at approximately a stipe length (cm) of 33.2 ± 14.8 SE (Fig. 4C and D).

### Mechanical testing

**Fig. 5:**
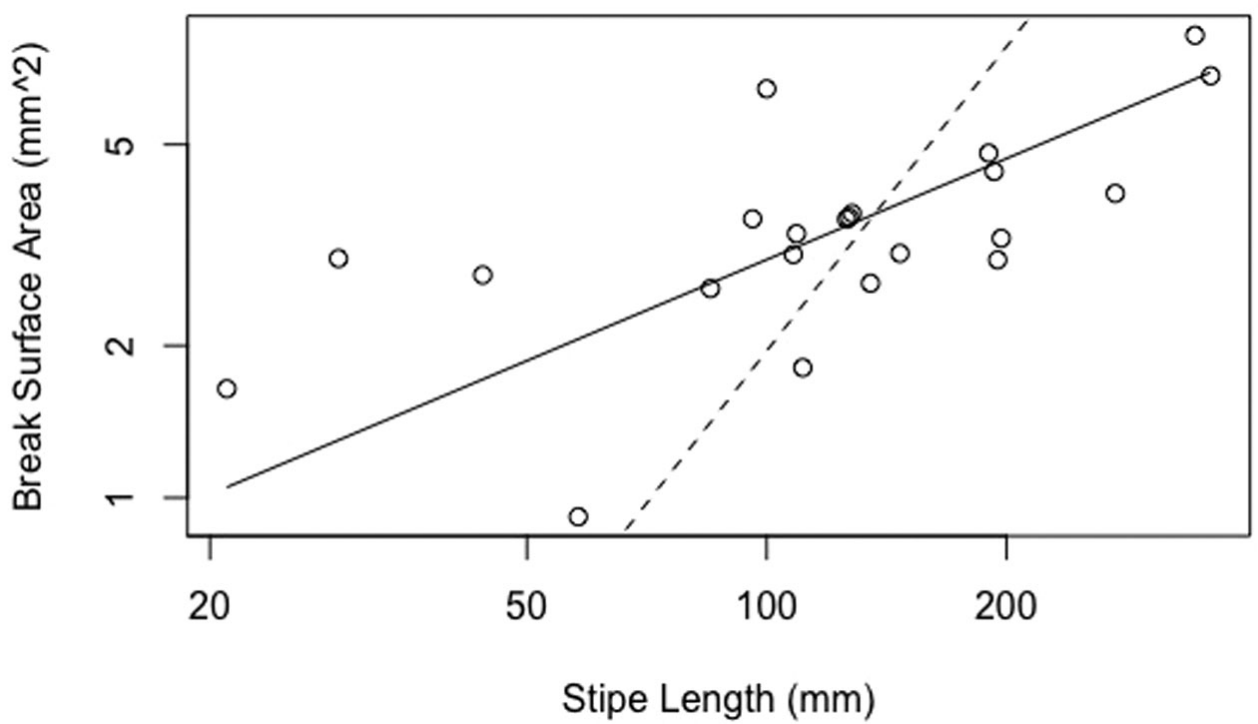
Surface area of stipe breaks increases hypoallometrically with stipe length. Solid lines show standard major axis regression lines; dotted lines show predicted isometry (predicted slope = *H*_0_ = 2).

**Fig. 6:**
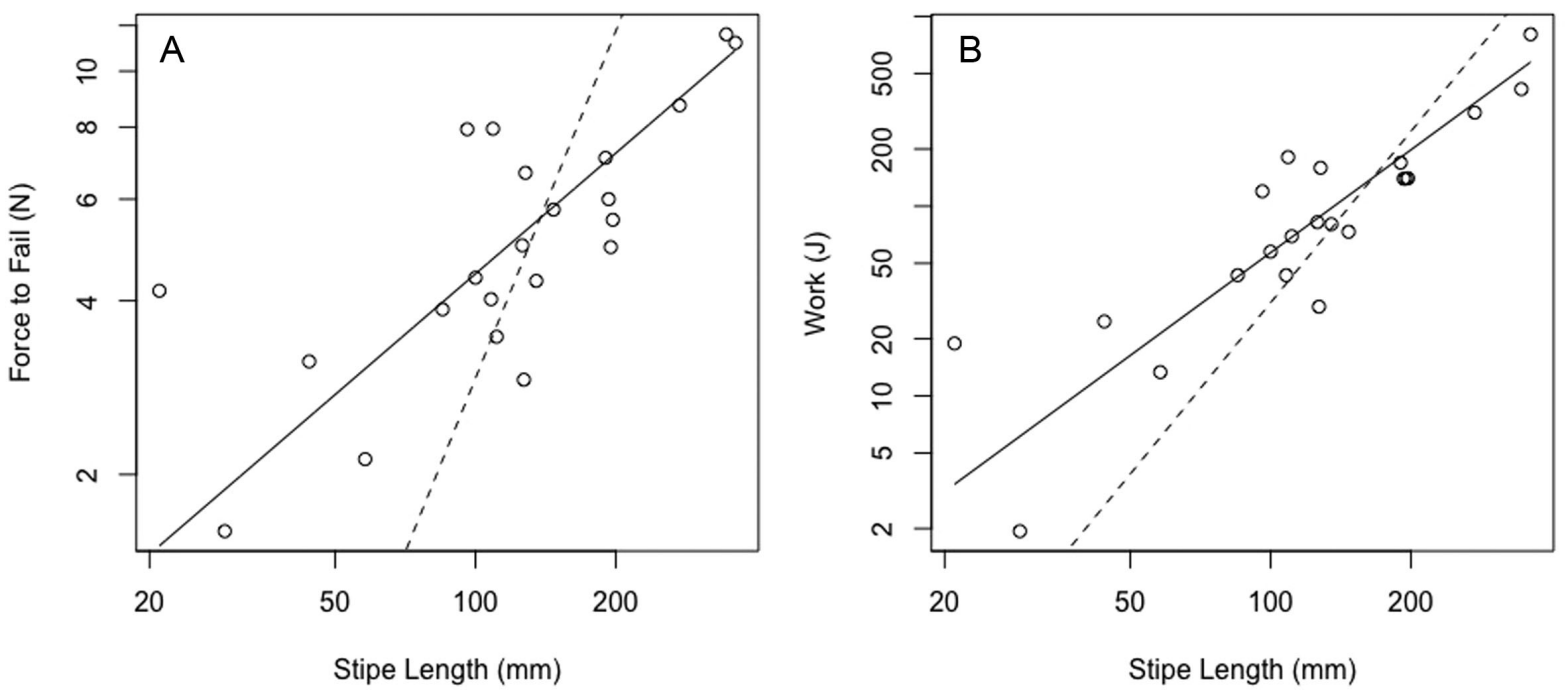
Both force to fail and W_fail_ increase less rapidly than stipe length. **(A)** F_fail_ scales with negative allometry (predicted slope = *H*_0_ = 2). **(B)** Work to failure scales with negative allometry (*H*_0_ = 3). Solid line shows standard major axis regression line; dotted line shows predicted isometry.

**Fig. 7:**
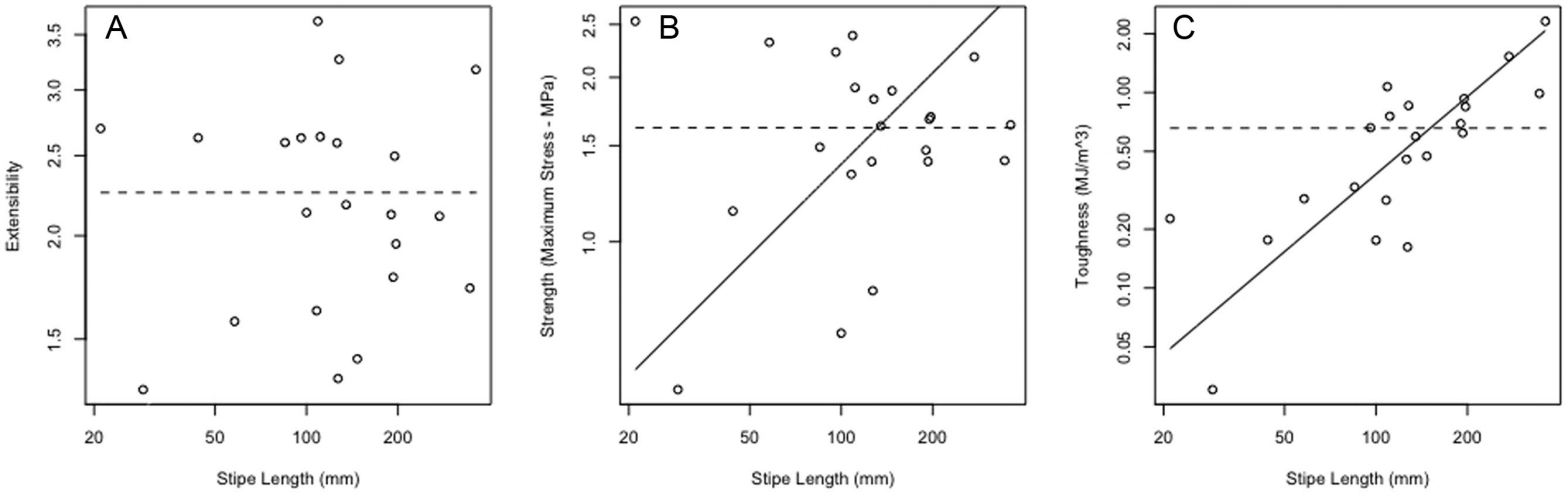
Extensibility is independent of stipe length, while strength (stress_max_) and toughness scale with positive allometry. **(A)** Extensibility (ε_fail_) does not change with stipe length (predicted slope = *H*_0_ = 0); dashed line shows predicted slope = *H*_0_ = 0. **(B)** Stress_max_ scales with positive allometry (predicted slope = *H*_0_ = 0). **(C)** Toughness (MJ m^−3^; predicted slope = *H*_0_ = 0) scales with positive allometry. On panels B and C, solid line shows standard major axis regression line; dotted line shows predicted isometry.

**Fig. 8:**
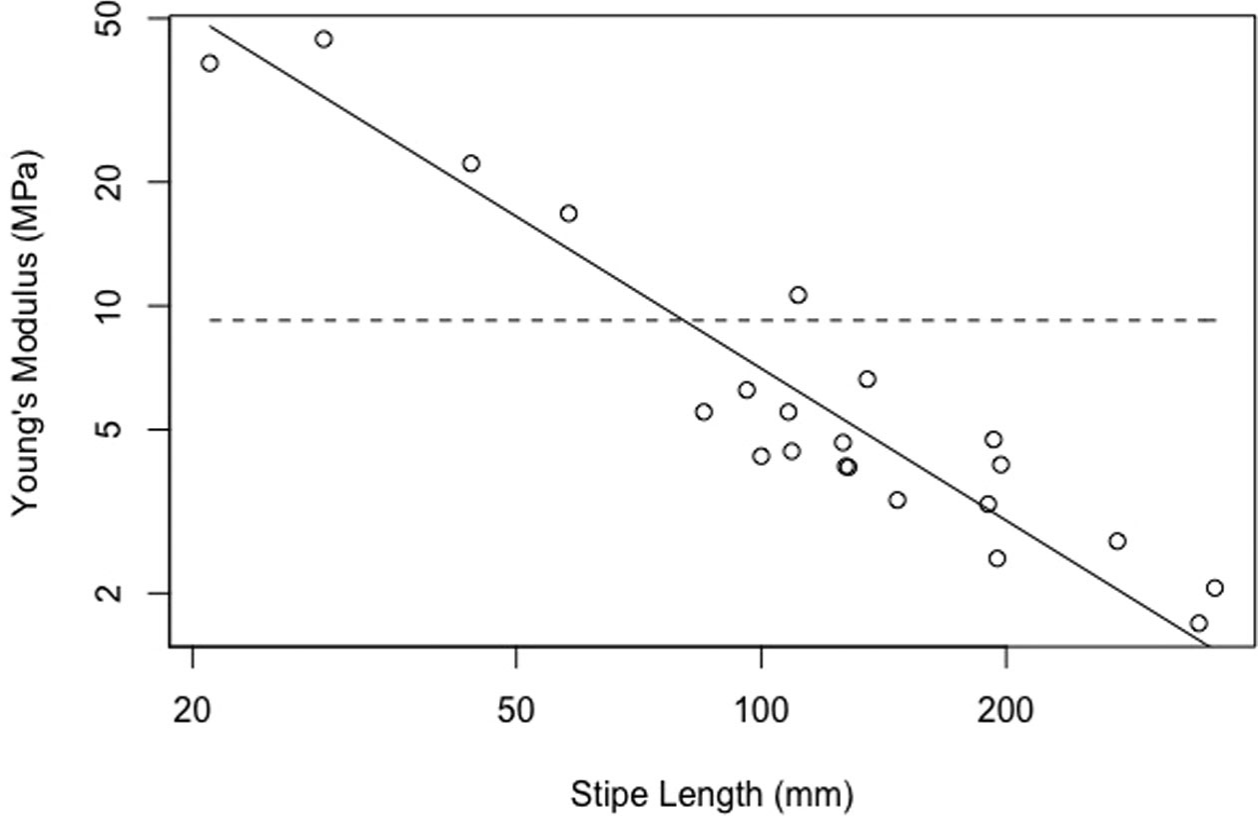
Young's modulus scales with negative allometry (predicted slope = *H*_0_ = 0).

Stipe break surface area shows negative allometry, increasing more slowly than stipe length (*b* = 0.66; Fig. 5). All stipes broke observably close to the bulb, but there was no statistically significant scaling relationship with overall stipe length (*R*^2 ^= 0.05). A comparison of F_fail_ versus stipe length displays negative allometry (slope = 0.69; Fig. 6A), as does W_fail_ (slope = 1.8; Fig. 6B). The slope of the regression line for ε_fail_ does not differ from 0 (slope = 0.02) and the *R*-squared value is nearly zero, indicating that ε_fail_, a measure of extensibility, is independent of stipe length (Fig. 7A). Contrary to our predictions, Young's modulus (slope = −1.23; Fig. 8), the σ_max_ (slope = 0.55; Fig. 7B), and toughness (slope = 1.31; Fig. 7C) do change over the course of bull kelp growth.

## Discussion

The rate of growth of *N. luetkeana* is not continuous during its annual growing season. The first few weeks of sporophyte growth (bulb diameter vs. stipe length; Fig. 4B) are isometric as the stipe grows from 2 cm to nearly 40 cm. However, we observed negative allometry in the full-grown bull kelp sporophyte, leading to relatively small bulbs (Fig. 4A; Denny et al. 1997). The observed breakpoint, between isometry and allometry, occurs when kelp stipes reach about 33 cm, after approximately one week of growth at a maximum growth rate of 6 cm per day, although there are many environmental factors that can influence growth rates (Scagel 1947); this value is very similar to the size of the largest juvenile bull kelp used in this study. The observed shift in scaling likely occurs because the early stages of bull kelp growth focus on increasing stipe length to reach the photic zone. Later, when the bulb and blades are nearer the surface, resources are allocated toward increasing blade surface area to maximize photosynthesis (Duncan and Foreman 1980).

Our results show that most *N. luetkeana* material properties change as juveniles grow. First, smaller kelp become tough and strong more rapidly than expected under isometry. This is in line with observations in other kelps, where younger tissues are less stiff than the older regions of the blade (Krumhansl et al. 2015).

However, the stiffness of the stipe in juvenile kelp follows an opposite pattern: the youngest stipes are stiffer than older stipes (Fig. 8; Fig. S1). This early focus on stiffness may be an adaptation to help resist damage from herbivores, since very small stipes are catastrophically harmed more easily (preventing further growth and development), whereas slightly older stipes can withstand higher levels of herbivore assault, but will be more vulnerable to multidirectional drag. In some perennial species, tissues may be stiffened by adding a thicker cortex during resting phases (Starko et al. 2018). However, bull kelp is an annual species, without a resting phase, and likely does not have this life history trait. As small bull kelp grow, they increasingly encounter variable flow regimes, where they experience drag forces from currents and surface winds, acting in multiple directions. Having the ability to reconfigure in response to these multidirectional forces to minimize damage becomes increasingly crucial compared with resisting herbivory in more mature bull kelp, since their size offers a refuge from consumers.

Finally, there was a little to no change in either extensibility (Fig. 7) or location of failure along the length of the stipe for juvenile kelp tested. Contrary to what might be expected based on kelp material properties, when stretched to a similar extent very small bull kelp stipes (length ≈ 2 cm) are no more or less likely to fail than larger juvenile bull kelp stipes (length ≈ 36 cm). This lack of pattern in extensibility is unexpected based on previous work showing older tissue in kelp, and red algae are both stiffer and less extensible than younger tissue (Krumhansl et al. 2015). While there was no significant pattern to the location of failure relative to stipe length, we consistently observed that stipes failed in areas of older tissue close to the bulb, rather than near the holdfast. Unlike the lack of change in extensibility, this is expected based on observed patterns of stiffness and strength in other species (Krumhansl et al. 2015; Burnett and Koehl 2019).

This similarity in extensibility, coupled with radically different environmental drag conditions, means that juvenile and mature kelp require different approaches to prevent stipe failure. Juvenile kelp exist closer to the substrate than mature kelp, where they likely will experience a very different flow regime. Mature bull kelp, with their bulbs floating near the surface, are known to experience peak tidal currents between 0.7 and 1.5 m s^–1^ at bulb level (Koehl and Wainwright 1977). Even within a few meters of the surface, maximum wave-generated speeds are two to three times the maximum current speed measured just above the substrate (Eckman et al. 2003). Not only are kelp near the benthos experiencing lower current speed, this disparity indicates that tidal currents are the primary source of drag near the benthos, meaning these kelp will experience much more unidirectional flows. To prevent stipe failure, kelp can mitigate the effects of high drag environments by passively reconfiguring their blade surface area, taking advantage of their tough, flexible stipe (Koehl and Wainwright 1977; Koehl and Alberte 1988). Because of the unidirectional, lower flow at the level of the benthos (Eckman et al. 2003), it is likely that younger juvenile kelp experience a relatively reduced drag regime with little need for such restructuring. In addition to this gross structural modification, more mature juvenile kelp also have overall stronger and tougher stipes than would be predicted by simple isometric growth. This is despite having a lower stiffness (Young's modulus) than would be predicted, indicating that both stipe structural and material properties change as bull kelp grow.

Factors other than drag may also influence juvenile bull kelp morphology and structural properties, such as grazing pressure. Even seemingly minor damage caused by mesograzers, like the snail (*L. vincta*), can have population-level impacts, making bull kelp more vulnerable to breakage (Duggins et al. 2001; Chenelot and Konar 2007). In a survey, stipes of individual, solitary drift bull kelp washed up on beaches appeared to have failed at locations of abrasion or damage from urchin feeding, likely due to a reduction in cross-sectional area (Koehl and Wainwright 1977). The shape of damage, in addition to the amount of damage, can also influence propagation of cracks in intertidal macroalgae and lead to increased likelihood of breakage (Mach et al. 2007). Such effects have not yet been studied in juvenile bull kelp sporophytes, which may be even more vulnerable due to their overall small cross-sectional stipe area and closer proximity to benthic grazers.

Mature *Nereocystis* sporophytes experience a vastly different environment than juvenile sporophytes. This is reflected in a host of adaptations, from the differences in scaling of juveniles and adults, to the scaling of material properties as kelp grow up through the water column. There is further work to be done to understand the variation between the biotic and abiotic pressures on juvenile and mature kelp, such as determining changes in predation pressure, the effects of biotic and abiotic damage on stipe failure, and changes in biomass allocation. This research highlights the importance of studying all stages of an organism's life cycle in order to account for differences in evolutionary pressures.

## Supplementary Material

obab022_Supplemental_File

## Data Availability

The data underlying this article have been uploaded to Dryad (https://doi.org/10.5061/dryad.t4b8gtj2b).
