# Supplementary figures and images for: Scaling and Structural Properties of Juvenile Bull Kelp (Nereocystis luetkeana)
Source: Integr Org Biol. 2021 Aug 14;3(1):obab022. doi: 10.1093/iob/obab022 (PMC8363980; doi:10.1093/iob/obab022)

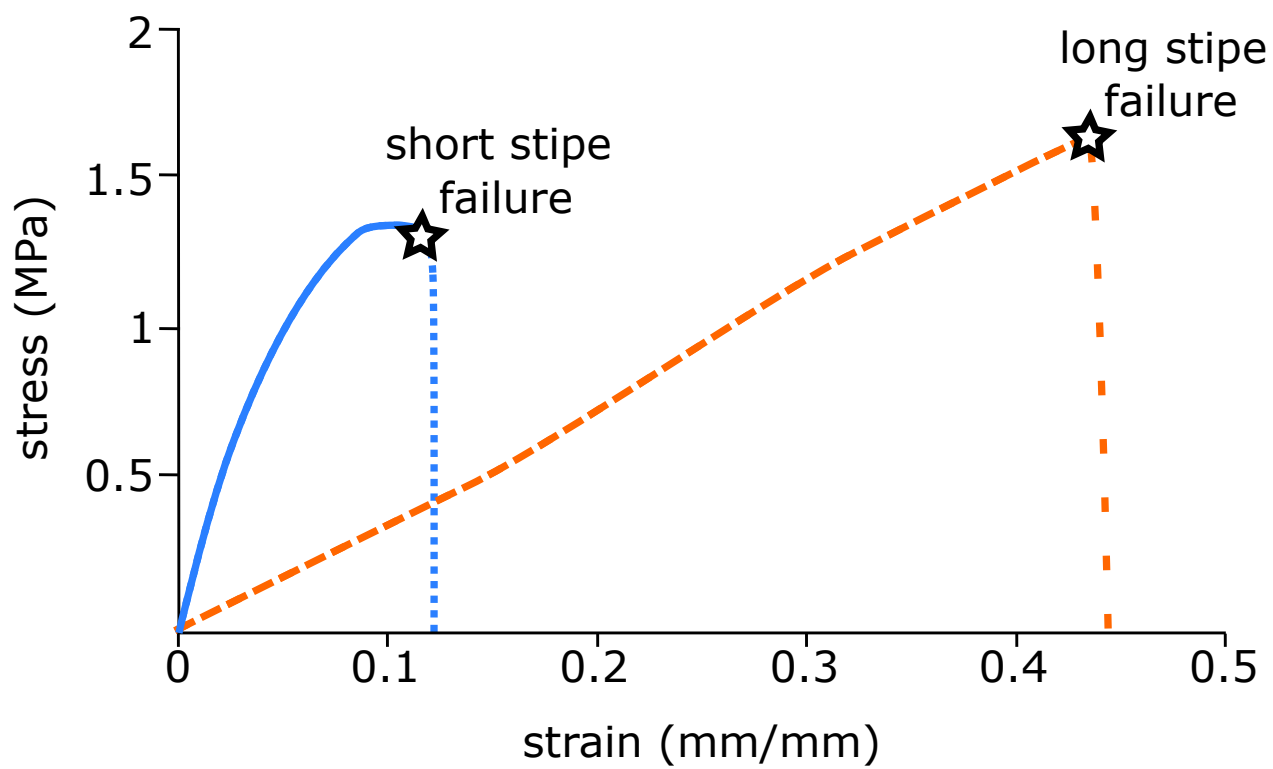

Supplement: obab022_Supplemental_File [file obab022_supplemental_file.pdf]
